# Impact of Annealing on Perpendicular Magnetic Anisotropy and Interfacial Diffusion in Ultrathin [CoFeB/Pd]×n Multilayer Film

**DOI:** 10.3390/nano16090558

**Published:** 2026-05-01

**Authors:** Lakshmanan Saravanan, Murugesan Praveen Kumar, Ayyanuservai Ravikumar, Govindhasamy Murugadoss, Roberto Rodríguez-Suárez, Smiljan Vojkovic, Delhibabu Prabhu, Shaik Gouse Peera, Carlos Garcia

**Affiliations:** 1 Departamento de Física, Universidad Técnica Federico Santa María, Valparaíso 2390123, Chile; 2 International Advanced Research Centre for Powder Metallurgy and New Materials, Balapur, Hyderabad 500005, India; prabhueins@gmail.com; 3 Department of Physics, J. J. College of Engineering and Technology, Trichy 620009, India; praveenkumarmurugesan280589@gmail.com; 4 School of Environment and Safety Engineering, Jiangsu University, Zhenjiang 212013, China; prmravikumar@gmail.com; 5 Centre of Excellence for Energy Research, International Research Centre, Sathyabama Institute of Science and Technology, Chennai 600119, India; 6 Facultad de Física, Pontificia Universidad Católica de Chile, Santiago 7820436, Chile; rrodrigueu@uc.cl (R.R.-S.); smiljan_87@hotmail.com (S.V.); 7 Natural Science Research Institute, College of Natural Sciences, Keimyung University, 1095 Dalgubeol-daero, Daegu 42601, Republic of Korea

**Keywords:** spintronics, magnetic thin films and multilayers, out-of-plane magnetic anisotropy, interfacial migration, anisotropic magnetoresistance, magnetoresonance

## Abstract

The multilayers of Ta/Pd/[CoFeB (0.3 nm)/Pd]×5/Pd films were fabricated by ultra-high-vacuum (UHV) magnetron sputtering and subsequently annealed at temperatures (T_A_) ranging from 100 °C to 400 °C. The magnetic measurements were performed with the applied field oriented parallel and perpendicular to the film plane to evaluate the out-of-plane magnetic anisotropy (PMA). A maximum effective PMA energy density (K_eff_) of ≈7.82 × 10^5^ erg/cc and a small out-of-plane saturation magnetisation (M_s⊥_) were achieved at the optimal T_A_. The evolution of PMA is associated with interfacial atomic migration and oxidation processes, as confirmed by X-ray photoelectron spectroscopy (XPS). Annealing at 300 °C initiates the formation of TaB and TaOB interfacial phases, whereas annealing at 400 °C promotes the enhanced growth of Ta_2_O_5_ and TaB, along with additional TaOB formation owing to increased oxygen migration. These thermally stable Ta–boride phases lead to pronounced modifications in the magnetic properties. Consequently, oxygen migration and interfacial reactions at elevated temperatures primarily alter the chemical states of the B 1s, Pd 3d, and Ta 4f orbitals, thereby influencing the PMA. The field-dependent electrical resistance (MR) study demonstrates that annealing at 100–400 °C optimises the anisotropic effect in the [CoFeB/Pd]×5-based multilayers. However, higher temperatures can trigger atomic intermixing, which degrades PMA strength and the resistance response. Moreover, the samples were further characterised by their structural, anomalous Hall effect (AHE) and magnetoresonance (MRO) properties. Overall, controlled T_A_-driven oxygen diffusion and interfacial oxidation enable effective tuning of the PMA, MR, and MRO properties of ultrathin [CoFeB/Pd]×5 multilayers, highlighting their strong potential for spin–orbit torque (SOT), Dzyaloshinskii–Moriya interaction (DMI), and magnetic skyrmion-based spintronic devices.

## 1. Introduction

Magnetic tunnel junctions (MTJs) with PMA are important because they offer high thermal stability and require lower switching current [1]. The switching process is governed primarily by spin-torque mechanisms, which depend on spin polarisation (P) and the materials used in the device structure. Recent research has focused on multilayer systems that use pure Co as the ferromagnetic (FM) layer. These layers are usually grown on Pt or Pd and are sometimes capped with Pd or Ta. Such nonmagnetic (NM)/FM/NM structures show strong PMA [2]. In recent years, CoFeB has become more popular than Co, Fe, and CoFe as the FM layer. This is because CoFeB offers better thermal stability, a high tunnel magnetoresistance (TMR) ratio, a high Curie temperature (T_c_), good P, moderate saturation magnetisation (M_s_), and low Gilbert damping (α). These properties enhance device speed and reduce power consumption by lowering the required switching current density [3]. However, the magnetic properties of CoFeB depend on its thermal treatment, thickness, and composition, so careful comparison of studies is needed [4]. For example, CoFeB with a 40:40:20 composition is often paired with Pt, Pd, or Ta layers, with MgO serving as a buffer, capping or interlayer [5,6]. If Ta is used without MgO, it can alter magnetic anisotropy and form a thick magnetic dead layer [7]. However, ultrathin CoFeB films deposited on Ta/Pd can still exhibit PMA even without annealing [8]. Very recently, PMA has also been reported in Co/Pd/Ta multilayer structures due to a thermal phase transformation [9].

Multilayer structures are important for MTJs used in non-volatile magnetic random-access memory (MRAM), improving device performance and reliability [10]. Synthetic ferromagnetic (SyF) structures consist of two or more FM layers separated by spacer layers. These structures help balance the net magnetic moment via the Ruderman–Kittel–Kasuya–Yosida (RKKY) interaction [11]. Additionally, SyF structures stabilise the reference layer (RL) during high-temperature back-end-of-line (BEOL) processing. A perpendicular [CoFeB/Pd] multilayer is widely used as both the RL and the free layer (FL). In modern MTJ systems, the Pd spacer layer plays a crucial role [12], and its thickness strongly influences magnetic-layer coupling. Furthermore, inadequate control of this thickness at elevated temperatures can cause interdiffusion at the interfaces, thereby reducing the exchange coupling strength. To achieve strong PMA in [CoFeB/Pd] systems, NM buffer and capping layers with a face-centred cubic (fcc) [Pd or Pt] structure are required. Pd is commonly used because it is cost-effective, supports PMA, and exhibits good thermal stability with CoFeB [8,12,13]. In contrast, Pt is more expensive but provides stronger coupling when used as a buffer, spacer, or capping layer [14]. Compared with common underlayers such as Cu, Au and Ti, Pd and Pt are used less often. However, they exhibit strong spin–orbit coupling (SOC). This increases spin pumping and raises the current required for spin-transfer torque (STT) switching. Even so, these materials are now widely studied for spin–orbit torque (SOT) switching. This method is promising for write operations in perpendicular MRAM devices [15]. Typical SOT devices use a multilayer structure comprising a heavy-metal (HM) layer, an FM layer, and an oxide layer. These devices require less switching energy than conventional STT-MRAM. The SOT effect arises from the strong SOC in HM materials such as Pt, Pd, Ta, and W. These materials generate a transverse spin current that exerts a torque on the magnetisation of the FM layer [16]. Therefore, systematic studies of perpendicular magnetic thin films with HM buffers and capping layers are important for the development of high-performance SOT-MRAM and next-generation spintronic devices.

A theoretical study using the variable-variance Preisach model accounted for the strong PMA observed in as-deposited Ta (2 nm)/Pd (2 nm)/[tCoFeB/tPd]×n multilayers. This work was reported by A. F. Franco et al. in 2016 [17]. Subsequently, A. F. Franco and colleagues studied ferromagnetic resonance, PMA, and interlayer exchange coupling in [CoFeB/Pd]×n and CoFeB/Pd/Co hard–soft structures in 2017 [18]. The findings indicated that PMA increases with the number of repetitions (n) and is affected by both interface quality and the FM layer’s thickness. In contrast, exchange coupling weakens as the CoFeB thickness increases [12]. These results demonstrate that each layer plays an important role in controlling magnetic properties. Recent studies have shown that [CoFeB/Pd]×n ultrathin multilayers exhibit strong PMA when the CoFeB thickness is less than or equal to 4 Å [12]. The maximum PMA is observed at 3 Å. For thicker layers, the magnetisation switches to the in-plane direction due to the competition between surface anisotropy and magnetoelastic effects. Effective damping decreases with thickness, reaching about 0.019 at 4 Å. Thus, these materials are suitable for high-speed, low-power microwave applications. From earlier reports, it is clear that the thicknesses of CoFeB and Pd, as well as n, are important parameters. The optimal values are 0.3 nm (3 Å) for CoFeB, 1 nm (10 Å) for Pd, and five repetitions, taken as a constant parameter for our work, because these conditions yield strong PMA and a distinct square hysteresis loop in the as-deposited state.

Annealing temperature (T_A_) is a critical parameter for enhancing PMA and promoting CoFeB crystallisation [19]. Numerous studies indicate that PMA can be achieved at T_A_ without a magnetic field [13,20,21]. Recent findings suggest that magnetic atoms primarily interact with oxygen [22,23]. Thus, precise control of oxygen migration, oxidation, and T_A_ is essential [24]. Noble metals with high oxygen affinity can effectively manage and redistribute oxygen at interfaces, particularly at CoFeB/Pd interfaces, which are especially effective in controlling interfacial oxygen behaviour [13,25]. Additionally, a low M_s_ value is important for low-power, high-speed spin switching in p-MTJs [26]. To date, the impact of T_A_ on PMA, interfacial oxidation, and diffusion within the Ta (2 nm)/Pd (2 nm)/[CoFeB (0.3 nm)/Pd (1 nm)]×5/Pd multilayer system has not been investigated. Therefore, a systematic study of how annealing affects these multilayers is necessary.

Here, we report for the first time the achievement of strong interfacial PMA with low M_s_ in Ta/Pd/[CoFeB(0.3 nm)/Pd(1 nm)]×5/Pd structures by optimising the T_A_. The results show that improved interfacial quality, driven by oxygen redistribution, is key to enhancing PMA and thermal stability. We also systematically investigate AHE, field-dependent electrical resistance, chemical states of the elements, magnetoresonance, and structural properties to understand the film’s behaviour in detail. These findings underscore the importance of precise interfacial control for optimising the performance of [CoFeB/Pd]×5 multilayers for spintronic and magnonic applications.

## 2. Materials and Methods

The Si//Ta(2 nm)/Pd(2 nm)/[CoFeB(0.3 nm)/Pd(1 nm)]×5/Pd(10 nm) stacks were deposited on native oxide Si (100) substrates at room temperature (RT) using a UHV magnetron sputtering system [AJA International, Inc., USA] with a base pressure higher than 5.1 × 10^−9^ Torr. During sputtering, high-purity 2” targets of Ta, Pd, and Co_40_Fe_40_B_20_ [CoFeB] (99.99%) were used, and the argon gas flow was maintained at 20 sccm. The sputtering powers and rates were 80 W (0.34 Å/s) for Ta, 40 W (0.78 Å/s) for Pd, and 40 W (0.16 Å/s) for CoFeB, all at a sputtering pressure of 3 mTorr. Pd and CoFeB were deposited using a direct current (DC) power supply, while Ta was deposited with a radio frequency (RF) power supply. The substrates rotated at 50 RPM to ensure film uniformity during deposition and to prevent the development of any in-plane magnetic anisotropy axes. Incorporating a Ta/Pd underlayer resulted in a smoother surface and better adhesion. This configuration further supports field-free switching of perpendicular magnetisation (Ta/Pd layers composed of two HM with opposite Spin Hall Angles), which is vital for SOT-MRAM applications [27]. A thick Pd capping layer protected the films against external oxidation and effectively shielded the underlying [CoFeB/Pd]×5 layers, thereby promoting good thermal stability. After deposition, Ex situ annealing was performed in the UHV main chamber at 1.8 × 10^−6^ Torr for 30 min at temperatures of 100–400 °C in the absence of an external magnetic field. The ramp-up rate was 2 °C/s, and the multilayer films were then removed from the high-vacuum chamber after cooling to RT.

The X-ray powder diffraction (XRD) patterns were obtained using a PANalytical X’pert PRO X-ray diffractometer with Cu K_α_ radiation (λ = 1.5406 Å). A glass holder was used to support the sample. The scanning range was 20° to 90° (2θ) with a step size of 0.02° and a step time of 1 s. The as-deposited film thickness of the multilayer was determined through X-ray reflectivity (XRR) using the same PANalytical X’pert PRO instrument, and the specular XRR spectra were fitted using the PANalytical X’pert software [X’Pert Reflectivity, v1.2a]. The AHE and electrical resistance were measured for the CoFeB-based multilayers at RT using a four-probe van der Pauw method with a magnetic field up to 1.0 T. A Keithley digital picoammeter [Model 6221] supplied a constant probe current of 1 mA and ensured a stable current source. At the same time, the voltage was measured with a Keithley digital voltmeter [Model 2700]. The ohmic nature of the contact was verified through these measurements. The in-plane (//) and out-of-plane (ꓕ) magnetic anisotropies of the multilayer films were evaluated using a homemade alternating gradient magnetometry (AGM) setup at RT. The interfacial oxidation states and binding energies of Co, Fe, B, Pd, Ta, O, and Si were analysed through XPS using a PHI 5000 VersaProbe III. An Al K_α_ X-ray source (1486.7 eV) was employed with a pass energy of 55 eV. The step profiling was carefully performed by varying the Agron gas etching time (time step: 60 s). The data were calibrated using the standard binding energy of the C 1s core level (284.8 eV). The XPSPEAK software (v4.1) was used to interpret the data and plot the analysis results. The electron paramagnetic resonance (EPR) experiments at RT were conducted using an X-band spectrometer (9.84 GHz) equipped with an electromagnet delivering a 1.0 T (DC) magnetic field [Bruker EMX-micro spectrometer]. The EPR measurements were conducted in two distinct geometries: in-plane and out-of-plane. Angle-dependent measurements were performed by rotating the film plane relative to a fixed magnetic field.

## 3. Results and Discussion

### 3.1. Anomalous Hall Effect (AHE) Analysis

**Figure 1:**
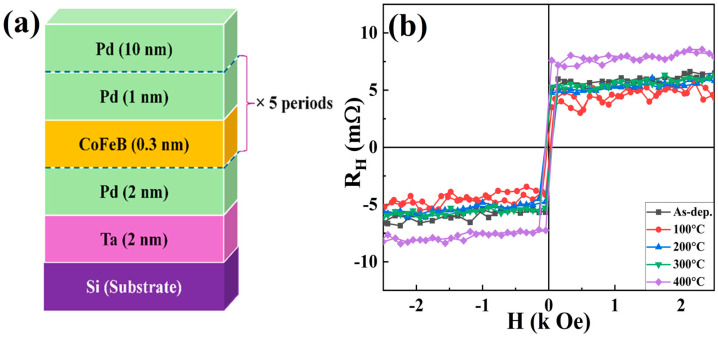
Description of the studied structures: (**a**) Schematic cross-section of the structure. (**b**) Field (out-of-plane) dependence of the Hall resistance in multilayers annealed at different temperatures.

Figure 1a shows a schematic of the multilayer structure: Ta (2.0 nm)/Pd (2.0 nm)/[CoFeB (0.3 nm)/Pd (1.0 nm)]×5/Pd (10 nm) deposited on a Si substrate. As shown in Figure 1b, the AHE results for the [CoFeB (0.3 nm)/Pd (1.0 nm)]×5 multilayer films annealed at various temperatures are presented. In general, a square-shaped hysteresis loop with no reduction in magnetisation at zero magnetic field is a strong indicator of PMA [8]. All samples annealed up to 400 °C exhibit a square loop, confirming interfacial PMA. These results demonstrate that PMA is significant across all samples and that the [CoFeB (0.3 nm)/Pd (1.0 nm)]×5 multilayer exhibits interfacial PMA at a higher T_A_. This may be a promising option for CMOS-compatible spintronic devices that require high thermal endurance and reliable Hall resistance performance. Previous studies have indicated that combining Ta with Pd or Pt as seed and capping layers improves the thermal robustness of PMA structures compared with Ta alone [12,13,27].

### 3.2. Field-Dependent Electrical Resistance Study

**Figure 2:**
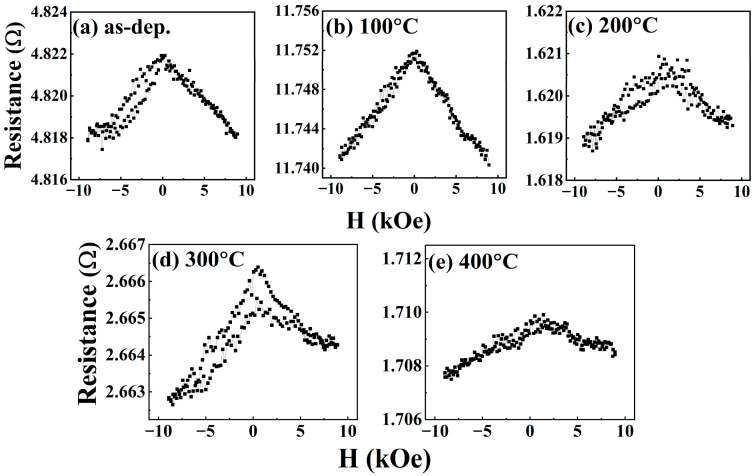
(**a**–**e**). R vs. H curves for Ta/Pd/[CoFeB(0.3)/Pd(1)]×5/Pd films prepared at different T_A_.

Figure 2 shows resistance (R) versus magnetic field (H) for Si//Ta/Pd/[CoFeB/Pd]×5/Pd multilayer thin films at various T_A_. All curves are symmetric, indicating the presence of anisotropic magnetoresistance (AMR). In this multilayer structure, ΔR is negative because resistance reaches a maximum at zero field and decreases as the magnetic domains align under the applied magnetic field. The as-deposited sample exhibits a bell-shaped curve with a resistance of 4.821 Ω. When annealed at 100 °C, the resistance increases to about 11.752 Ω, which could be attributed to initial structural changes and increased interfacial scattering [28,29,30]. At 200 °C, the resistance drops sharply to 1.620 Ω due to improved crystallinity and reduced defects. After annealing at 300 °C, the resistance increases slightly to 2.666 Ω with sharper peaks, indicating a more pronounced domain-wall effect [31,32]. Upon further annealing at 400 °C, the resistance peak broadens and flattens. This indicates the degradation of PMA due to Pd (1 nm) atoms migrating into the ultra-thin CoFeB (0.3 nm) layers (a Pd diffusion event) or to atomic intermixing, which destroys the discrete FM structure and forms a diluted magnetic alloy or a magnetic dead layer at the interfaces [30]. These results indicate that 200–300 °C is the optimal annealing range for enhancing magnetic anisotropy in this multilayer system.

Moreover, the strength of PMA varies with T_A_ because it is strongly affected by interfacial oxidation, diffusion, and structural disorder. These factors adversely affect electrical resistance at higher temperatures. The next section will thoroughly explore the presence of interfacial PMA.

### 3.3. Perpendicular Magnetic Anisotropy Investigations

**Figure 3:**
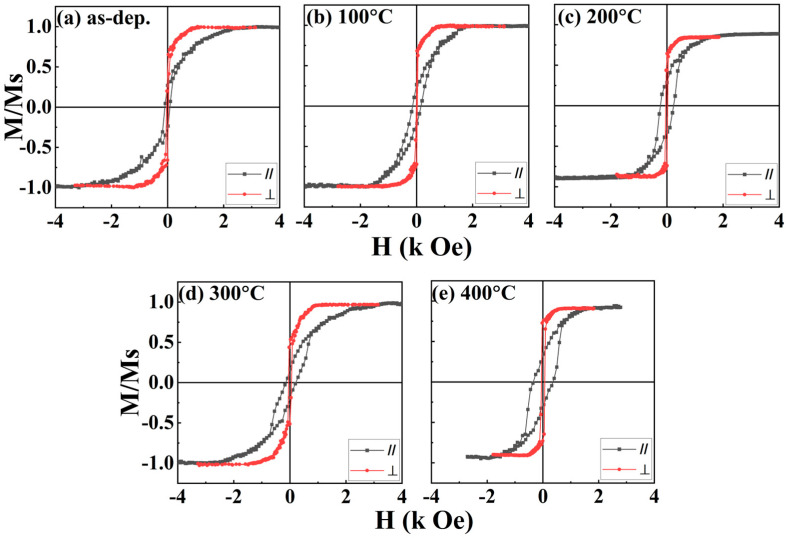
Normalised in-plane and out-of-plane M-H loops of CoFeB-based multilayer films at different temperatures: as-dep. (**a**), T_A_ = 100 °C (**b**), T_A_ = 200 °C (**c**), T_A_ = 300 °C (**d**) and T_A_ = 400 °C (**e**). For better comparison, the magnetisation is presented in normalised form (M/M_s_).

The AGM was used to detect interfacial PMA in multilayer films at RT. The magnetic field was applied either parallel (in-plane) or perpendicular (out-of-plane) to the Ta(2)/Pd(2)/[CoFeB(0.3)/Pd(1)]×5/Pd(10) stacks. Previous studies have shown that T_A_ and magnetic layer thickness are critical parameters that influence multilayer systems [30]. In particular, the effect of T_A_ on PMA is a key factor in optimising emerging magnetic structures. Accordingly, our primary objective is to achieve robust PMA in these novel multilayer stacks. Figure 3a–e show the magnetic hysteresis loops measured along both the in-plane and out-of-plane directions for the Ta/Pd/[CoFeB(0.3)/Pd(1)]×5/Pd films under different T_A_ conditions. In all cases, the samples exhibit an easy axis aligned with the out-of-plane direction, indicating the presence of PMA in the films.

In general, the motion of very thin Bloch-type domain walls tends to favour PMA. By contrast, thicker Néel-type domain walls are usually associated with IPA [33]. In this study, interfacial PMA is attributed solely to the presence of Bloch-type domain walls, which are observed in all samples. These Bloch-type domain walls are likely formed under higher thermal annealing conditions (up to 400 °C), which significantly modify interfacial PMA. Therefore, our research indicates that magnetic anisotropy (PMA strength) in Ta/Pd/[CoFeB(0.3)/Pd(1)]×5/Pd stacks is mainly influenced by T_A_, the thickness of the FM layer, the choice of HM, and the overall multilayer structure design.

**Figure 4:**
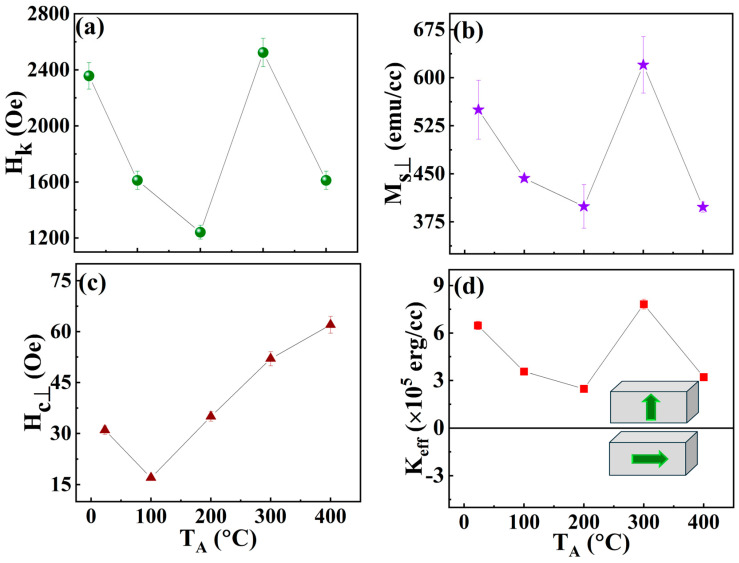
Variation of (**a**) H_k_, (**b**) M_sꓕ_, (**c**) H_cꓕ_, and (**d**) K_eff_ with T_A_ up to 400 °C.

From the magnetic hysteresis curves in Figure 3, the H_k_ values for the Ta/Pd/[CoFeB(0.3)/Pd(1)]×5/Pd multilayers were determined to be 2.35, 1.61, 1.24, 2.52 and 1.61 kOe for the as-dep., 100, 200, 300 and 400 °C samples, respectively. The variation in H_k_ with T_A_ is shown in Figure 4a. The positive and negative values indicate out-of-plane (PMA) and in-plane (IPA) magnetic anisotropies, respectively. This analysis shows that higher H_k_ values correspond to stronger magnetic anisotropy in the multilayer stacks, which is directly influenced by thermal annealing. Interfacial PMA is observed across all samples (from as-dep. to 400 °C) and underscores the importance of thermal annealing for achieving robust PMA.

**Figure 5:**
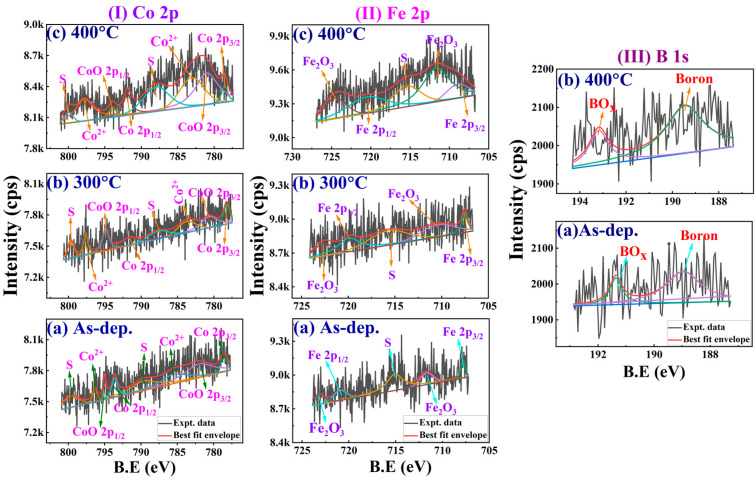
XPS core-level spectra for (**I**) Co 2p and (**II**) Fe 2p—[(**a**) as-dep., (**b**) 300 °C and (**c**) 400 °C] and (**III**) B 1s—[(**a**) as-dep., and (**b**) 400 °C] for Ta/Pd/)/[CoFeB(0.3 nm)/Pd(1.0 nm)]×5/Pd films. The ‘S’ labels represent the presence of satellite peaks.

**Figure 6:**
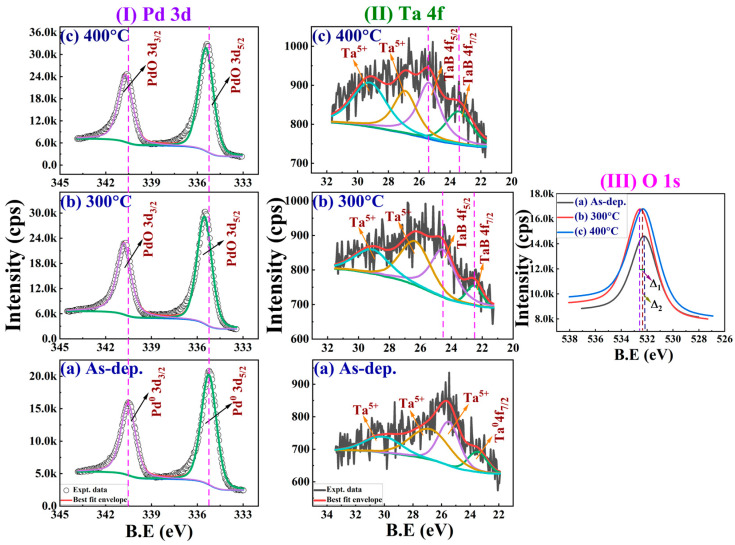
Core-level XPS spectra illustrating (**I**) Pd 3d, (**II**) Ta 4f, and (**III**) O 1s, captured before (**a**) as-dep. and following annealing at (**b**) 300 °C and (**c**) 400 °C for Ta/Pd/[CoFeB(0.3)/Pd(1)]×5/Pd multilayer films.

Figure 4b shows a gradual decrease in M_s⊥_ as T_A_ increases from RT (as-dep.) to 200 °C. However, upon annealing at 300 °C, M_s⊥_ increases sharply in the [CoFeB/Pd]×5 system. With a further increase in T_A_ to 400 °C, M_s⊥_ declines rapidly. The decrease in M_s⊥_ probably stems from a thinner CoFeB layer due to intermixing with the buffer (Pd) layers [34] along with increased oxidation at the Pd/CoFeB and CoFeB/Pd/Ta interfaces [13]. This indicates that at higher T_A_ (400 °C), oxygen can diffuse into the Pd, Ta, and CoFeB layers. Additionally, it is well known that hybridisation between B 1s and CoFe 3d states reduces the magnetic moments of Co and Fe and induces a small negative magnetic moment in boron atoms [35]. The observed increase in M_s⊥_ can be due to a reduction in the number of boron atoms, which are considered magnetic impurities in the amorphous CoFeB film during annealing [36]. According to the rigid band model, the magnetic moment of CoFe (a transition-metal alloy) increases as the concentration of boron (a metalloid) decreases. This behaviour is explained by boron’s much smaller atomic size compared with elements such as Pd, Co, Fe, and Ta, which makes boron diffusion more efficient. Consequently, its redistribution during annealing significantly influences the magnetic properties of the multilayer film. Our results indicate that magnetisation (specifically M_s⊥_) is significantly affected by the presence of CoO and FeO, as well as by oxidation at interfaces between PdO and TaO_x_ layers. These oxidation processes are confirmed by XPS (Figure 5 and Figure 6) and by XRD. The influence of oxygen diffusion and interfacial oxidation on magnetisation is consistent with findings reported in recent studies [37,38].

The H_c⊥_ for all the samples at various temperatures is shown in Figure 4c. The as-dep. sample initially exhibits a higher H_c⊥_ value (31 ± 02 Oe). After annealing at 100 °C, H_c⊥_ decreases to 17 ± 01 Oe and then gradually increases to 62 ± 03 Oe at 400 °C. According to the uniform rotation model of magnetisation reversal, an optimal PMA sample should have a significant H_c⊥_ value, and thus the optimal temperature for the largest H_c⊥_ is 400 °C. Nevertheless, variations in H_c⊥_ may be attributed to changes in the magnetic pinning sites in the multilayer films [37]. The out-of-plane saturation magnetisation, coercivity, anisotropy field, squareness ratio, and anisotropy energy density of the multilayers are measured at RT, 100 °C, 200 °C, 300 °C, and 400 °C, as shown in Table 1.

The K_eff_ value is determined as follows [39]:

(1) $$K_{eff}=\frac{M_{S}\times H_{K}}{2}$$

The K_eff_ is generally separated into contributions from volume anisotropy (K_v_) and interface anisotropy (K_i_). The equation below usually describes K_eff_ [40].

(2) $$K_{eff}=K_{V}-2\pi M_{S}^{2}+\frac{K_{i}}{t_{CoFeB}}$$

Here, $2\pi M_{S}^{2}$ and $t_{CoFeB}$ are the demagnetisation energy and the thickness of the magnetic layer (CoFeB film), respectively. Equation (2) indicates that when K_eff_ > 0, i.e., when the out-of-plane magnetic anisotropy exceeds the demagnetising energy, the easy axis of magnetisation tends to align perpendicular to the film plane. Conversely, when K_eff_ < 0, the films’ magnetic easy axis promotes alignment in the in-plane/parallel plane. Figure 4d shows the T_A_ dependence of K_eff_ in Ta/Pd/[CoFeB(0.3)/Pd(1)]×5/Pd heterostructure films, providing additional information on the interfacial PMA of CoFeB films. As the T_A_ is tuned, the magnetic anisotropy of the samples shifts between in-plane and out-of-plane, demonstrating that the interface is critical to PMA at the [CoFeB(0.3)/Pd(1)]×5 interfaces on the Ta/Pd buffer layers. The K_eff_ initially decreases with T_A_ from RT to 200 °C, then increases to a maximum of 7.824 ± 0.312 × 10^5^ erg/cc at 300 °C. Beyond this point, K_eff_ decreases again at 400 °C. This maximum K_eff_ value is lower than the PMA energy (2.1 × 10^6^ erg/cc) determined in MgO (001)//Pt(10 nm)/[CoFeAl0.5Si0.5/Pt]×n/Pt(10 nm) multilayers [41]. Nevertheless, the recorded K_eff_ value is significantly higher than that of previously reported Ta or Al/Pd(20)/[CoFeB(0.3)/Pd(0.8)]×N stacks on different substrates, which have a maximum K_eff_ value of 5.6 × 10^5^ erg/cc [42]. Therefore, these Si(100)//Ta(2 nm)/Pd(2 nm)/[CoFeB(0.3 nm)/Pd(1.0 nm)]×5/Pd(10 nm) multilayer stacks are highly promising candidates for novel spintronic devices.

### 3.4. Interfacial Oxidation and Diffusion Analysis

XPS is a highly effective and widely used method for investigating T_A_ effects on the development of interfacial oxidation states and atomic motion. The redistribution and/or diffusion of oxygen atoms at the interfaces render the oxidation states highly sensitive to thermal treatment [13]. Conversely, the magnetic anisotropy of the films is strongly influenced by interlayer diffusion and oxidation at the FM/HM interfaces. We performed XPS measurements on selected as-dep. and annealed Ta/Pd/[CoFeB(0.3)/Pd(1)]×5/Pd multilayers. The results confirm the presence of Pd, Co, Fe, B, Ta and O in the stacks.

Figure 5I,II show the core-level XPS spectra of Co 2p and Fe 2p for both as-dep. and annealed Ta (2 nm)/Pd (2 nm)/[CoFeB (0.3 nm)/Pd (1.0 nm)]×5/Pd (10 nm) multilayer films. The metallic Co 2p_3/2_ and 2p_1/2_ peaks appear at ≈778.5 and ≈793.6 eV, respectively [43]. Peaks corresponding to cobalt oxide (CoO_x_) are observed at around 781.8 and 795.0 eV. As shown in Figure 5I, both Co and CoO_x_ signals were detected in all three sample conditions: as-dep. [Figure 5I(a)], annealed at 300 °C [Figure 5I(b)], and at 400 °C [Figure 5I(c)]. The Co 2p_3/2_ and 2p_1/2_ peaks align well with values reported in previous studies [13,22,37]. Notably, all samples exhibit a strong Co-O bonding signature with two dominant peaks at binding energies of ≈785.3 eV (Co^2+^ 2p_3/2_) and 796.3 eV (Co^2+^ 2p_1/2_). Figure 5II(a–c) presents the fitted Fe 2p spectra. Iron oxide (Fe_2_O_3_) is indicated by peaks at ≈711.6 and ≈723.2 eV, while metallic Fe 2p_3/2_ and 2p_1/2_ peaks appear at around 707.8 and 720.8 eV, respectively. The oxidised forms of Co and Fe are more prominent in the annealed samples than in the as-dep. sample, suggesting the coexistence of both metallic (Co and Fe) and oxidised (Co–O and Fe–O) bonding states. Additionally, the Co and Fe contents in the CoFeB layer vary with T_A_, and both elements exhibit signs of oxidation.

Figure 5III shows the core-level B 1s spectra of the as-dep. and annealed samples at different temperatures. Two distinct peaks are observed, corresponding to two chemical states of B 1s: (i) B from the CoFeB film, and (ii) BO_x_. These findings are consistent with previous reports [13,37]. In the as-dep. sample [shown in Figure 5III(a)], a strong B 1s signal appears at ≈188.9 eV, indicating the presence of metallic boron. This peak reflects covalent bonding between boron and transition metals (B–CoFe compounds). An additional oxidised B 1s peak (BO_x_) is observed at ≈191.4 eV and is attributed to the natural oxidation of boron atoms near the interface [44]. Upon annealing, the metallic boron peak at ≈188.9 eV remains nearly unchanged at 300 °C and shifts to ≈ 189.4 eV at 400 °C [in Figure 5III(b)], accompanied by a notable increase in intensity. Meanwhile, the BO_x_ peak remains at ≈ 191.4 eV at 300 °C but shifts to ≈ 193.2 eV at 400 °C and increases in intensity. This shift is attributed to boron’s higher affinity for oxygen than Pd at elevated temperatures. During annealing, boron migrates from the CoFeB layer towards the adjacent Pd layer, where it competes with Pd for available oxygen [13]. As a result, both the binding energies and intensities of the metallic boron and BO_x_ peaks remain relatively unchanged between the as-dep. and 300 °C samples. At 400 °C, the binding energy of metallic boron increases by 0.5 eV, whereas that of BO_x_ increases by 1.8 eV. Their peak intensities also rise by 3.3 and 1.2%, respectively. These trends are consistent with observations in CoFeB/MgO/CoFeB stacks, where both metallic boron and BO_x_ states evolve during annealing [37,45]. The broadening of the BO_x_ spectrum is attributed to the formation of B_2_O_3_ and boron suboxides [45]. The increased concentration of BO_x_ in the CoFeB layer is due to oxygen incorporation during annealing at elevated temperatures [37,45].

From Table 2, we infer that annealing at 300 °C significantly increases oxidation at the interfaces, whereas at 400 °C more atomic interdiffusion and oxidation occur extensively, which could significantly degrade the magnetic and electrical transport properties.

The XPS spectra of Pd for both the as-deposited and annealed samples are shown in Figure 6I(a–c). The two orbital components (Pd 3d_5/2_ and Pd 3d_3/2_) are identified as the characteristic peaks of Pd. To investigate interactions within the Ta/Pd/[CoFeB (0.3 nm)/Pd (1.0 nm)]×5/Pd multilayers, depth profiling was performed up to ≈30 nm. In the as-deposited sample [Figure 6I(a)], strong metallic Pd (Pd^0^) peaks appear at binding energies of ≈335.2 eV (Pd 3d_5/2_) and ≈340.5 eV (Pd 3d_3/2_). This interpretation is further supported by previous reports [13,46]. Upon annealing at 300 °C [Figure 6I(b)], these peaks shift to higher energies [≈335.55 eV and ≈340.8 eV], indicating the slight oxidation of Pd. The binding energy and intensity changes from the as-deposited state are ~0.3 eV and 36%, respectively. At 400 °C [Figure 6I(c)], the Pd^0^ peaks shift slightly to lower energies by about 0.11 eV. At the same time, the reduced oxidised Pd signal suggests significant oxygen diffusion into the multilayer. Compared with the 300 °C sample, the Pd 3d_5/2_ and Pd 3d_3/2_ peak intensities increase by 8.51% and 7.44%, respectively, indicating changes in oxidation states and Pd distribution after annealing. Additionally, the 300 °C sample likely forms a highly oxidised transition layer at the Pd/CoFeB interface. In contrast, at 400 °C, Pd oxide levels decrease despite increased BO_x_ content, implying that oxygen is consumed by boron to form stable boron oxides. These observations support the conclusion that boron’s competing affinity for oxygen influences the evolution of Pd’s oxidation states during thermal treatment.

The XPS spectra of Ta in both as-dep. and annealed samples are shown in Figure 6II(a–c). In the as-dep. film, both metallic and oxidised states of Ta were observed. A metallic Ta^0^ 4f_5/2_ peak appears at ≈23.5 eV, consistent with previous studies [13,47]. The presence of Ta oxides was expected because the film [Ta layer] was deposited directly onto the native oxide of the Si substrate, which facilitated TaO_x_ formation through interaction with oxygen. Both the as-dep. and annealed samples (at 300 °C and 400 °C) show clear peaks corresponding to Ta_2_O_5_ [Ta in the +5 oxidation state]. This observation aligns with Pauling’s electronegativity scale: Ta (1.5) has a significantly lower electronegativity than Fe (1.83), Co (1.88), B (2.04), Pd (2.2), and O (3.44), thereby confirming its strong affinity for oxygen [44,48]. Moreover, a thicker Pd capping layer effectively prevented external oxidation of the underlying Ta, Pd, and CoFeB layers at RT. In the as-dep. sample [in Figure 6II(a)], distinct Ta 4f peaks were observed at ≈23.50 eV (Ta^0^ 4f_5/2_), ≈25.52 eV (Ta_2_O_5_ 4f_7/2_), and ≈27.01 eV (Ta_2_O_5_ 4f_5/2_). After annealing at 300 °C, additional peaks at ≈22.51 and ≈24.55 eV appeared and were attributed to the formation of TaB [44] as shown in Figure 6II(b). Simultaneously, the Ta_2_O_5_ peaks shifted to lower binding energies (~26.40 and 29.15 eV) and are likely due to the formation of mixed oxide–boride compounds (TaOB) [44]. At 400 °C [in Figure 6II(c)], further shifts in the XPS spectra were observed. The Ta_2_O_5_ 4f_7/2_ and 4f_5/2_ peaks shifted to ≈26.93 and ≈29.28 eV, respectively. Meanwhile, the intensities of the TaB 4f_7/2_ (~23.42 eV) and 4f_5/2_ (~25.42 eV) peaks decreased. The increase in binding energy of these boride peaks suggests the enhanced formation of TaOB and a slight increase in Ta_2_O_5_ content at 400 °C. The increasing intensity of the Ta_2_O_5_ peak at ≈29.28 eV indicates that the oxide layer thickens during prolonged annealing. This temperature also promotes boron diffusion from the CoFeB layer into the Ta layer, thereby facilitating TaB formation. Concurrently, boron incorporation into the Ta_2_O_5_ matrix likely forms Ta–O–B bonds, yielding TaOB [13,44,49]. The formation of TaB and TaOB introduces compositional changes at the interface, which can affect magnetic properties and depend on the sharpness of the interface. Among transition metal borides, the enthalpies of formation are Co–B (−32 kJ/mol), Fe–B (−36 kJ/mol), and Ta–B (−78 kJ/mol). The more negative Ta–B enthalpy indicates superior thermodynamic stability. The enhanced Ta–B peak observed here likely indicates that the Ta–boride layer becomes dominant over the CoFe–boride layer. This is consistent with prior studies [13].

Oxygen migration and redistribution make interfacial oxidation highly dependent on the T_A_ [44]. Figure 6III(a–c) shows the O 1s XPS spectra of Ta/Pd/[CoFeB (0.3 nm)/Pd (1 nm)]×5/Pd multilayer films in their as-dep. state and after annealing at 300 °C and 400 °C. A single O 1s peak is observed across all samples, and the as-dep. film shows a peak at ≈532.20 eV. After annealing at 300 °C [shown in Figure 6III(b)], the O 1s peak shifts by ~0.35 eV to higher binding energy and increases in intensity by 14.15%, which indicates a greater involvement of oxygen at the interface. When annealed further at 400 °C, the peak moves slightly to a lower binding energy (~532.34 eV) [Figure 6III(c)]. This indicates changes in oxygen’s chemical environment. The energy shifts (Δ_1_ and Δ_2_) reflect changes in the number of oxygen vacancies induced by heating. The drop in binding energy at 400 °C may result from oxygen redistribution or redox-driven interfacial diffusion at the interfaces. At this temperature, slower oxygen release favours the formation of oxides of Co, Fe, B, Pd, and Ta. As a result, the BO_x_ and TaO_x_ signals remain stable even at higher temperatures. At the same time, boron diffuses into the Ta layer and forms TaB, which mixes with metallic Ta (Ta^0^) upon further heating [44]. In conclusion, the changes in the O 1s, Co 2p, Fe 2p, and Pd 3d spectra with temperature show clear oxidation and oxygen movement patterns at 300 °C and 400 °C. At 400 °C, increased oxygen diffusion into the Ta, Pd, and CoFeB layers alters the interfaces and weakens the PMA properties.

### 3.5. Magnetic Resonance Investigations

**Figure 7:**
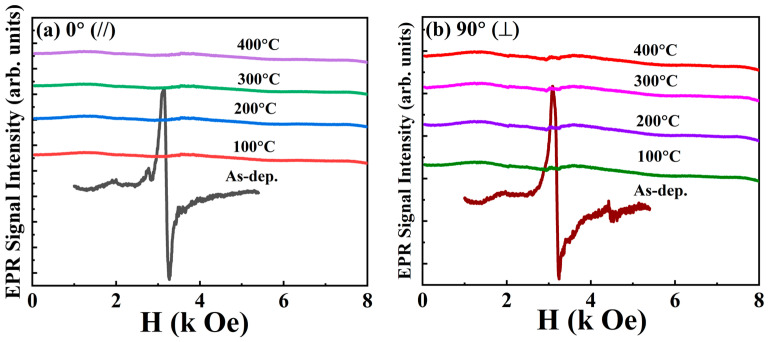
Dependence of the magnetic resonance spectra on the (**a**) in-plane and (**b**) out-of-plane angles for the as-dep. and annealed (100–400 °C) Ta/Pd/[CoFeB(0.3)/Pd(1)]×5/Pd multilayer.

The EPR is a valuable technique for investigating magnetic properties such as the g-factor, spin relaxation time, magnetic anisotropy constant and effective magnetisation [50]. Figure 7a,b display the magnetic resonance spectra measured at 0° and 90° for samples subjected to various T_A_. A noticeable EPR signal is observed in the as-dep. film, indicating relatively low magnetic anisotropy. However, this signal vanishes after annealing, suggesting a transformation in magnetic behaviour in which FMR becomes dominant due to increased magnetic ordering. Similar behaviour has been previously observed in CoTa thin films, where post-annealing leads to the disappearance of the EPR signal and the appearance of FMR-related magnetic anisotropy [50]. For the annealed [CoFeB/Pd]×5 multilayers, the absence of the EPR spectrum could be attributed to the following possibilities [51,52]: (1) stronger magnetic exchange interactions, (2) modifications in magnetic anisotropy, (3) faster spin-lattice relaxation processes, (4) structural changes such as crystallisation and interlayer diffusion, and (5) a decrease in the concentration of paramagnetic centres.

Finally, the absence of the EPR signal in annealed [CoFeB/Pd]×5 multilayers is primarily due to the transition from a paramagnetic (PM) to an FM state. In the FM state, strong exchange interactions and magnetic order cause significant line broadening, rendering the EPR signal undetectable. Annealing also induces interdiffusion and/or oxidation at the CoFeB/Pd interfaces, altering the local magnetic environment and reducing the number of unpaired spins. Additionally, Boron diffusion from the CoFeB layers during annealing promotes crystallisation, allowing CoFe(B) to align with the bcc MgO structure [53]. This structural transition reduces the number of PM centres and enhances FM ordering. Together, these magnetic and structural changes account for the suppression of the EPR signal after annealing.

**Figure 8:**
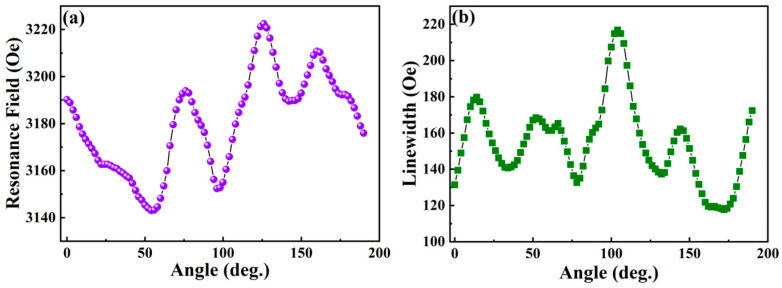
Angular dependence of the (**a**) resonance field and (**b**) linewidth measured for the as-dep. CoFeB-type multilayer films at 300 K, exhibiting 180° periodicity.

Due to demagnetisation effects, the resonance field of the multilayer film varies with its orientation relative to the magnetic field direction, even in the PM phase. For example, Figure 8a shows the angular dependence of the resonance field measured at 300 K.

The magnetic anisotropy of the resonance field position and the EPR signal intensity exhibits clear oscillatory behaviour. The EPR linewidth also shows a similar oscillatory trend as the angle of orientation changes. The typical angular dependence of the EPR linewidth for the as-dep. [CoFeB/Pd]×5 multilayer is shown in Figure 8b. Both the EPR field and the linewidth depend on the orientation of the magnetic field relative to the specimen’s crystallographic axes.

### 3.6. Crystallographic Structure Analysis

**Figure 9:**
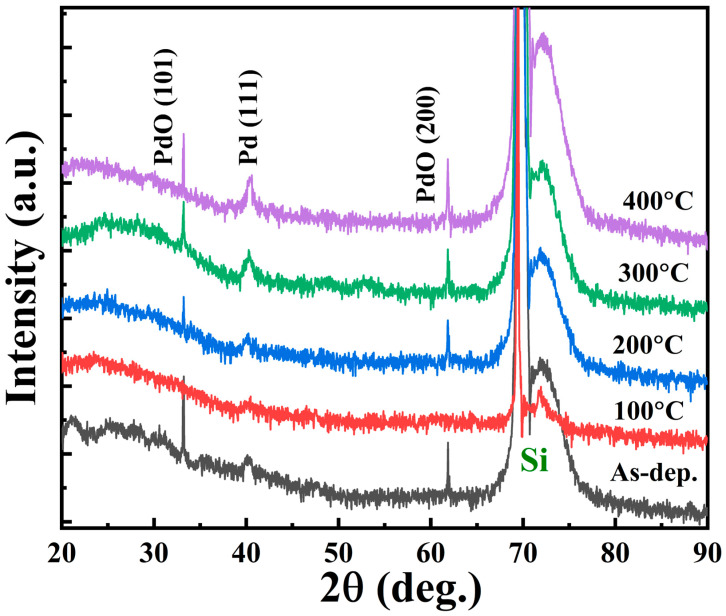
XRD patterns of Ta (2 nm)/Pd (2 nm)/[CoFeB (0.3 nm)/Pd (1 nm)]×5/Pd (10 nm) multilayer films deposited on a silicon substrate. The diffraction profiles are shown for the as-dep. sample and after annealing at 100, 200, 300 and 400 °C.

To examine the crystalline structures of CoFeB, Pd, and Ta films in multilayer stacks, GI-XRD analyses were performed on both as-dep. and annealed Si//Ta (2 nm)/Pd (2 nm)/[CoFeB (0.3 nm)/Pd (1 nm)]×5/Pd (10 nm) samples (shown in Figure 9). The measurements were conducted at a fixed glancing angle of 0.7°. For both the as-dep. and annealed conditions, two prominent diffraction peaks were observed: one attributed to Pd and PdO (expected due to the 10 nm-thick Pd capping layer used in our case), and the other corresponding to the (400) reflection of the Si substrate. Phase identification was performed using the ICDD-JCPDS database [54].

Despite these observations, no significant changes in the crystallisation of the CoFe(B) layers were detected in the XRD patterns. This is likely due to the extremely thin nature of the CoFe(B) films and the resolution limitations inherent in standard laboratory XRD systems. High-resolution transmission electron microscopy (HR-TEM) is suggested as a complementary technique to provide a more detailed understanding of the crystalline quality of these multilayers. Further experimental work is needed to uncover the microscopic origins of these structural variations.

## 4. Conclusions

This study investigates the effects of T_A_ on the magnetic, spin-transport, spin-resonance, interfacial oxidation/migration, and structural properties of Ta(2 nm)/Pd(2 nm)/[CoFeB(0.3 nm)/Pd(1 nm)]×5/Pd(10 nm) multilayers. The highest effective PMA energy density (K_eff_) of ≈7.82 × 10^5^ erg/cc is achieved at T_A_ = 300 °C, with a small M_s⊥_ of ≈620 emu/cc. Field-dependent resistance measurements indicate that 200–300 °C is the optimal annealing range for enhancing magnetic anisotropy in this multilayer system. Above 400 °C, Pd diffusion, magnetic dead-layer formation, over-oxidation, and interfacial mixing can degrade resistance and PMA. However, PMA remains noticeable at a higher T_A_ of 400 °C. XPS analysis shows that oxides and borides form and evolve at different interfaces, and that boron helps remove oxygen at high temperatures. The presence of Ta–boride (TaB) phases and oxidation-driven interfacial changes significantly influence magnetic and spin-transport behaviour. Both AGM and EPR measurements were used to gain a more detailed understanding of the magnetic anisotropy of the as-dep. and annealed stacks.

Finally, this work shows that strong PMA is observed in both the as-deposited and annealed films. The 300 °C sample exhibits enhanced PMA due to optimal interfacial oxidation in the Ta (2 nm)/Pd (2 nm)/[CoFeB (0.3 nm)/Pd (1 nm)]×5/Pd (10 nm) multilayers. This also yields a 20.7% improvement in PMA energy compared with the as-deposited film. In contrast, further annealing at 400 °C significantly degrades magnetic anisotropy and electrical transport properties due to atomic intermixing and excessive interfacial oxidation. Therefore, these findings underscore the importance of precise interfacial control and highlight the critical role of oxidation in [FM/HM]×n-based structures for enhancing interfacial PMA. We believe this approach offers a promising pathway for future spintronic device applications.

From a technological perspective, the Ta(2 nm)/Pd(2 nm)/[CoFeB(0.3 nm)/Pd(1 nm)]5/Pd(10 nm) multilayer offers high-density data storage, rapid switching and good thermal stability for nanoelectronic devices. Annealing enables precise control of PMA, M_s_, interfacial diffusion and/or oxidation. The ultrathin CoFeB layer maximises interfacial effects and the interface-to-volume ratio, while oxygen migration fine-tunes the magnetic properties. These characteristics demonstrate the structure’s potential for spintronic and neuromorphic applications and support the development of advanced memory technologies that address the memory wall challenges in AI hardware and energy-efficient data centres [55].

## Figures and Tables

**Table 1:** Out-of-plane magnetic properties of [CoFeB(0.3)/Pd(1)]×5-based structures after annealing at varying temperatures.

S. No	T_A_ (°C)	Magnetic Properties				
		M_sꓕ_ (emu/cc)	H_cꓕ_ (Oe)	H_k_ (Oe)	M_r_/M_s_	K_eff_ (×10^5^ erg/cc)
1	As-dep.	550 ± 46	31 ± 02	2357 ± 94	0.56	6.481 ± 0.259
2	100	443 ± 05	17 ± 01	1612 ± 64	0.50	3.570 ± 0.142
3	200	399 ± 34	35 ± 02	1242 ± 50	0.73	2.477 ± 0.099
4	300	620 ± 44	52 ± 02	2524 ± 101	0.51	7.824 ± 0.312
5	400	398 ± 08	62 ± 03	1612 ± 64	0.78	3.207 ± 0.128

**Table 2:** Summary table of XPS after peak fitting oxidation percentage of Co, Fe, B, Pd, Ta and O spectra for the selected Ta/Pd/[CoFeB(0.3)/Pd(1)]×5/Pd multilayers before annealing (as-dep.) and after annealing at 300 °C and 400 °C.

Multilayer Structure	CoO (2p_3/2_)		FeO (2p_3/2_)		BO_x_		PdO (3d_5/2_)		TaO (4f_5/2_)		O	
	Pos. (eV)	Conc. (%)	Pos.(eV)	Conc. (%)	Pos.(eV)	Conc. (%)	Pos.(eV)	Conc. (%)	Pos.(eV)	Conc. (%)	Pos. (eV)	Conc. (%)
As-dep.	781.8	76	711.6	41	191.4	40	-	-	27.01	73	532.20	30
300 °C	780.6	80	710.2	68	191.4	46	335.55	60	26.41	80	532.55	35
400 °C	780.9	38	711.5	47	193.2	16	335.44	51	26.93	44	532.34	32

## Data Availability

The original contributions presented in this study are included in the article. Further inquiries can be directed at the corresponding authors.

## Abbreviations

The following abbreviations are used in this manuscript:

UHV	Ultra-High-Vacuum
PMA	Perpendicular Magnetic Anisotropy
XPS	X-ray Photoelectron Spectroscopy
MR	Magnetoresistance

## References

[1] Ikeda S., Miura K., Yamamoto H., Mizunuma K., Gan H.D., Endo M., Kanai S., Hayakawa J., Matsukura F., Ohno H. (2010). A perpendicular-anisotropy CoFeB–MgO magnetic tunnel junction. Nat. Mater..

[2] Huang K.-F., Wang D.-S., Lin H.-H., Lai C.-H. (2015). Engineering spin-orbit torque in Co/Pt multilayers with perpendicular magnetic anisotropy. Appl. Phys. Lett..

[3] Wang M., Zhang Y., Zhao X., Zhao W. (2015). Tunnel Junction with Perpendicular Magnetic Anisotropy: Status and Challenges. Micromachines.

[4] Zhu M., Chong H., Vu Q.B., Brooks R., Stamper H., Bennett S. (2016). Study of CoFeB thickness and composition dependence in a modified CoFeB/MgO/ CoFeB perpendicular magnetic tunnel junction. J. Appl. Phys..

[5] Akyol M. (2019). Origin of interfacial magnetic anisotropy in Ta/CoFeB/MgO and Pt/CoFeB/MgO multilayer thin film stacks. J. Supercond. Nov. Magn..

[6] Jung J.H., Lim S.H., Lee S.R. (2010). Strong perpendicular magnetic anisotropy in an MgO/CoFeB/Pd unit structure with a thick CoFeB layer. J. Appl. Phys..

[7] Vardhana H., Srihari V., Sharma K., Singh S., Gupta M., Reddy V., Das S., Gome A., Gupta A., Sharma G. (2023). Ta interfaced CoFeB: Role of CoFeB thickness and thermal annealing in modification of structural and magnetic properties. Surf. Interfaces.

[8] Ngo D.-T., Quach D.-T., Tran Q.-H., Møhave K., Phan T.-L., Kim D.-H. (2014). Perpendicular magnetic anisotropy and the magnetization process in CoFeB/Pd multilayer films. J. Phys. D Appl. Phys..

[9] Rawat K., Sharma A., Brajpuriya R.K., Reddy V., Gloskovskii A., Kaushik V., Gupta A., Pathak S. (2025). Enhanced perpendicular magnetic anisotropy through thermal phase transformation in Co/Pd/Ta multilayers. Appl. Surf. Sci..

[10] Sato H., Ikeda S., Fukami S., Honjo H., Ishikawa S., Yamanouchi M., Mizunuma K., Matsukura F., Ohno H. (2014). Co/Pt multilayer based reference layers in magnetic tunnel junctions for nonvolatile spintronics VLSIs. Jpn. J. Appl. Phys..

[11] Bruno P., Chappert C. (1991). Oscillatory coupling between ferromagnetic layers separated by a nonmagnetic metal spacer. Phys. Rev. Lett..

[12] Silva A.S., Sá S.P., Bunyaev S.A., Garcia C., Sola I.J., Kakazei G.N., Crespo H., Navas D. (2021). Dynamical behaviour of ultrathin [CoFeB (tCoFeB)/Pd] films with perpendicular magnetic anisotropy. Sci. Rep..

[13] Lakshmanan S., Romanque C., Mery M., Muthuvel M.R., Gupta N.K., Garcia C. (2024). Effects of interfacial oxygen diffusion on the magnetic properties and thermal stability of Pd/CoFeB/Pd/Ta heterostructure. J. Alloys Compd..

[14] Li M., Yang H., Xie Y., Huang K., Pan L., Tang W., Bao X., Yang Y., Sun J., Wang X. (2023). Enhanced Stress Stability in Flexible Co/Pt Multilayers with Strong Perpendicular Magnetic Anisotropy. Nano Lett..

[15] Brink A.v.D., Vermijs G., Solignac A., Koo J., Kohlhepp J.T., Swagten H.J.M., Koopmans B. (2016). Field-free magnetization reversal by spin-Hall effect and exchange bias. Nat. Commun..

[16] Song C., Zhang R., Liao L., Zhou Y., Zhou X., Chen R., You Y., Chen X., Pan F. (2021). Spin-orbit torques: Materials, mechanisms, performances, and potential applications. Prog. Mater. Sci..

[17] Franco A.F., Gonzalez-Fuentes C., Morales R., Ross C.A., Dumas R., Åkerman J., Garcia C. (2016). Variable variance Preisach model for multilayers with perpendicular magnetic anisotropy. Phys. Rev. B.

[18] Franco A.F., Gonzalez-Fuentes C., Akerman J., Garcia C. (2017). Anisotropy constant and exchange coupling strength of perpendicularly magnetized CoFeB/Pd multilayers and exchange springs. Phys. Rev. B.

[19] Liu Y., Hao L., Cao J. (2016). Effect of annealing conditions on the perpendicular magnetic anisotropy of Ta/CoFeB/MgO multilayers. AIP Adv..

[20] Sinha J., Hayashi M., Kellock A.J., Fukami S., Yamanouchi M., Sato H., Ikeda S., Mitani S., Yang S.-H., Parkin S.S.P. (2013). Enhanced interface perpendicular magnetic anisotropy in Ta|CoFeB|MgO using nitrogen doped Ta underlayers. Appl. Phys. Lett..

[21] Meng H., Lum W.H., Sbiaa R., Lua S.Y.H., Tan H.K. (2011). Annealing effects on CoFeB-MgO magnetic tunnel junctions with perpendicular anisotropy. J. Appl. Phys..

[22] Rawat K., Sharma A., Brajpuriya R.K., Reddy V., Gloskovskii A., Vardhan H., Gupta A., Kaushik V., Pathak S. (2026). Depth selective magnetization investigation in thermally reduced Co/Pd multilayers revealed by MCD study. Appl. Surf. Sci..

[23] Wang Z.C., Saito M., McKenna K.P., Fukami S., Sato H., Ikeda S., Ohno H., Ikuhara Y. (2016). Atomic-Scale structure and local chemistry of CoFeB–MgO magnetic tunnel junctions. Nano Lett..

[24] Hassan M., Laureti S., Bergenti I., Spizzo F., Del Bianco L., Gnoli L., Barucca G., Ullrich A., Mezzi A., Albrecht M. (2026). On the origin of perpendicular magnetic anisotropy in STO/Co/X (X=Pd, Pt) sputtered films. Appl. Surf. Sci..

[25] Gilbert D., Olamit J., Dumas R., Kirby B.J., Grutter A.J., Maranville B.B., Arenholz E., Borchers J.A., Liu K. (2016). Controllable positive exchange bias via redox-driven oxygen migration. Nat. Commun..

[26] Butler W.H., Mewes T., Mewes C.K.A., Visscher P.B., Rippard W.H., Russek S.E., Heindl R. (2012). Switching Distributions for Perpendicular Spin-Torque Devices Within the Macrospin Approximation. IEEE Trans. Magn..

[27] Han F., Du W., Liu M., Su H., Zhang H., Liu B., Meng H., Tang X. (2022). Effects of Ta and Pt/Ta seed layer on the thermal stability of CoFeB/MgO perpendicular magnetic anisotropy film. J. Alloys Compd..

[28] Karahan İ.H., Bakkaloğlu Ö.F., Bedir M. (2007). Giant magnetoresistance of electrodeposited Cu-Co-Ni alloy films. Pramana.

[29] Zhang Y.Q., Zhang Z.D., Xiao Q.F., Geng D.Y., Zhao X.G., Zhang W.S., You C.Y. (2003). Giant magnetoresistance of Co Ni Cu alloys produced by mechanical alloying. J. Phys. D Appl. Phys..

[30] An G.G., Lee J.B., Yang S.M., Kim J.H., Chung W.S., Yoon K.S., Hong J.P. (2015). Correlation between Pd metal thickness and thermally stable perpendicular magnetic anisotropy features in [Co/Pd]n multilayers at annealing temperatures up to 500 °C. AIP Adv..

[31] Truong Q.D. (2019). Magnetic properties and domain structure of CoFeB/Pd multilayers with perpendicular magnetic anisotropy. Vietnam. J. Sci. Technol..

[32] Quach D.-T., Chu T.-D., Ngo D.-T., Kim D.-H. (2020). Correlation of hysteresis loop and domain structure of CoFeB/Pd multilayer at various temperatures. Phys. B.

[33] Ngo D.-t., Meng Z.L., Tahmasebi T., Yu X., Thoeng E., Yeo L.H., Rusydi A., Han G.C., Teo K.-l. (2013). Interfacial tuning of perpendicular magnetic anisotropy and spin magnetic moment in CoFe/Pd multilayers. J. Magn. Magn. Mater..

[34] Yamanouchi M., Koizumi R., Ikeda S., Sato H., Mizunuma K., Miura K., Gan H.D., Matsukura F., Ohno H. (2011). Dependence of magnetic anisotropy on MgO thickness and buffer layer in Co20Fe60B20-MgO structure. J. Appl. Phys..

[35] Tanaka H., Takayama S., Hasegawa M., Fukunaga T., Mizutani U., Fujita A., Fukamichi K. (1993). Electronic structure and magnetism of amorphous Co1−xBx alloys. Phys. Rev. B Condens. Matter.

[36] Wang Y.-H., Chen W.-C., Yang S.-Y., Shen K.-H., Park C., Kao M.-J., Tsai M.-J. (2006). Interfacial and annealing effects on magnetic properties of CoFeB thin films. J. Appl. Phys..

[37] Saravanan L., Gupta N.K., Pandey L., Kokila I.P., Therese H.A., Chaudhary S. (2021). Observation of uniaxial magnetic anisotropy and out-of-plane coercivity in W/Co20Fe60B20/W structures with high thermal stability. J. Alloys Compd..

[38] You C.Y., Fu H.R., Zhang X., Tian N., Wang P.W. (2014). Interaction of Ta–O and perpendicular magnetic anisotropy of Ta/Pd (0–2.4 nm)/Co2FeAl0.5Si0.5/MgO/Ta structured films. J. Magn. Magn. Mater..

[39] Johnson M.T., Bloemen P.J.H., den Broeder F.J.A., de Vries J.J. (1996). Magnetic anisotropy in metallic multilayers. Rep. Prog. Phys..

[40] Saravanan L., Manivel Raja M., Prabhu D., Pandiyarasan V., Ikeda H., Therese H.A. (2018). Impact of MgO thickness on the perpendicular magnetic anisotropy of Mo/Co2FeAl/MgO/Mo multilayers with improved annealing stability. Mater. Res. Bull..

[41] Wu Y., Zhang J., Wang Z.C., Wang J., Xu X.G., Miao J., Zhang J.X., Jiang Y. (2014). Perpendicular magnetic anisotropy and thermal stability in Co2FeAl0.5Si0.5/Pt multilayers. Appl. Phys. A.

[42] Jung J.H., Jeong B., Lim S.H., Lee S.-R. (2010). Strong perpendicular magnetic anisotropy in CoFeB/Pd multilayers. Appl. Phys. Express.

[43] Moulder J.F., Chastain J. (1992). Handbook of X-Ray Photoelectron Spectroscopy: A Reference Book of Standard Spectra for Identification and Interpretation of XPS Data, Update.

[44] K S.S., Gupta N., Perumal H.P., Kumar D., Gupta M., Gupta P., Sinha J. (2023). Interfacial electronic structure modulated magnetic properties in Ta/CoFeB/Ta multilayers. Surf. Interfaces.

[45] Niwa M., Yasui A., Ikenaga E., Honjo H., Ikeda S., Nakamura T., Endoh T. (2019). Change in chemical bonding state by thermal treatment in MgO-based magnetic tunnel junction observed by angle-resolved hard X-ray photoelectron spectroscopy. J. Appl. Phys..

[46] Pillo T., Zimmermann R., Steiner P., Hüfner S. (1997). The electronic structure of PdO found by photoemission (UPS and XPS) and inverse photoemission (BIS). J. Phys. Condens. Matter.

[47] Panigrahi B., Sahoo S.K., K S.S., Sinha J., Basumatary H., Raja M.M., Haldar A. (2022). Effect of Ta capping layer on spin dynamics in Co50Fe50 thin films. Solid State Commun..

[48] Lakshmanan S., Rao S.K., Muthuvel M.R., Chandrasekaran G., Therese H.A. (2017). Variable substrate temperature deposition of CoFeB film on Ta for manipulating the perpendicular coercive forces. J. Magn. Magn. Mater..

[49] Sato S., Honjo H., Ikeda S., Ohno H., Endoh T., Niwa M. (2015). Evidence of a reduction reaction of oxidized iron/cobalt by boron atoms diffused toward naturally oxidized surface of CoFeB layer during annealing. Appl. Phys. Lett..

[50] Topkaya R. (2017). Effect of post-annealing on structural and magnetic properties of CoTa alloy thin films. J. Supercond. Nov. Magn..

[51] Baberschke K. (2011). Ferromagnetic resonance in nanostructures, rediscovering its roots in paramagnetic resonance. J. Phys. Conf. Ser..

[52] Jhajhria D., Pandya D.K., Chaudhary S. (2018). Interplay of composition and anisotropy on evolution of microstructural, static and dynamic magnetic properties of CoFeB thin films on annealing. J. Alloys Compd..

[53] Kim J.-S., Kim G., Jung J., Jung K., Cho J., Kim W.-Y., You C.-Y. (2022). Control of crystallization and magnetic properties of CoFeB by boron concentration. Sci. Rep..

[54] Reddy G.K., Ling C., Peck T.C., Jia H. (2017). Understanding the chemical state of palladium during the direct NO decomposition–influence of pretreatment environment and reaction temperature. RSC Adv..

[55] Ranjan A., Farooq T., Chi C.-C., Wang C.-C., Lin Y.-L., Padilla R.I.S., Li Y.-H., Tseng Y.-C., Lee C.-H., Lu M.-Y. (2026). Stack-Engineered Mode Selection in PtMn/(Co/Pd)_n_ Multilayers Enables Deterministic Analog Spin–Orbit Torque Synapses. ACS Appl. Mater. Interfaces.

